# Digital Dependence in Organizations: Impacts on the Physical and Mental Health of Employees

**DOI:** 10.2174/17450179-v19-e230109-2022-17

**Published:** 2023-01-25

**Authors:** Lucio Lage Gonçalves, Antonio Egidio Nardi, Anna Lucia Spear King

**Affiliations:** 1 Av. Jornalista Ricardo Marinho, 150, ap. 1404 - Barra da Tijuca - Rio de Janeiro - Brasil – CEP 22631 350, Brazil

**Keywords:** Digital dependence, Digital addiction, Internet dependence, Digital impacts, Digital human behavior, Digital addiction in organizations, Digital addiction of employees

## Abstract

Digital Dependence is a person's persistent inability to regulate digital devices on which they have become highly dependent. Internet dependence has been described since the mid-1990s, and studies on this topic have intensified since 2010. This type of individual dependence has received considerable published literature, but it is new in the collective setting of organizations, offering the hypothesis that it can also be collective, given the impacts it can provide. Research has evolved geographically from three countries to 17 since the beginning of the last decade, with 7 new scales for digital dependence. There were 13 new revalidations of the Nomophobia Questionnaire (NMP-Q), with an increase from 1,000 to 13,000 volunteers. Geographical evolution and an increase in the number of scales and volunteers and their different profiles were described. New approaches reinforce evolution and its impacts on human behavior. This study provides historical insight into Digital Dependence and opens new prospects for research on the differences between nations and people, sexes, professionals, and the need for further research in organizations.

## 1 INTRODUCTION

With the growing competitiveness in organizations, digital transformation, and the COVID-19 pandemic, digital resources have intensified their use. With the pandemic, working from home office due to social isolation, people started to use them even more, breaking boundaries between work and rest, invading individual and family leisure time.

Social isolation tends to provoke psychological reactions, such as increased levels of anxiety, stress, and irritability, propitiate the appearance of fears (based on real or subjective information), and confused thinking, negatively affecting the individual's ability to make coherent decisions [1].

Many organizations, even before the pandemic, were using home offices from the facilities of applications used via digital devices to manage, run and expand their business. This strategy has been annihilating the traditional 8-hour workday, making employees reach their tasks in the same measure as at home, on the street, in entertainment, or at any time. There are no boundaries between this journey, fun, and interpersonal coexistence, making the collective environment a phenomenon of digital dependence.

### 1.1 Impacts

Impacts on human behavior are relevant when changes in the routine of individuals occur, requiring changes in habits and ways of relating, especially when conditions of isolation and excessive use are imposed.

### 1.2 In the Physical and Mental Health

During the last decade, research on “addictive technological behaviors” has grown substantially, demonstrating a strong association between the addictive use of technology and comorbid psychiatric disorders. Cross-sectional research with 23,533 individuals confirmed the relationship between symptoms of attention deficit (ADHD), obsessive-compulsive disorder (OCD), anxiety, and depression could explain the variance in the addictive use of two types of modern online technologies: social networks and video games [2].

Digital Ergonomics needs to be observed, as physical damage related to improper postures and incorrect furniture when using devices from digital work at home can cause damages [3, 4].

The improper handling of computers, cell phones, and tablets, among others, has favored the appearance of pathologies and functional physical limitations observed more frequently in medical offices [3, 5].

The phototoxicity of violet light from digital devices can lead to progressive degeneration of the macula, a noble area of ​​vision, which can cause irreversible damage to individuals exposed continuously and prolonged to this luminosity [3, 6].

Neuropsychological and neuroimaging research into excessive and addictive Internet use is a rapidly growing scientific field. It has revealed results of scientific and clinical impact and helped to understand the neurobiological basis of Internet addiction, converging on the view that addictive internet use is linked to functional brain changes involving parts of the prefrontal cortex, accompanied by changes in others. Cortical regions such as temporal and subcortical. These results suggest that prefrontal control processes are reduced in Internet-addicted individuals and may be related to patients' loss of control over this use [7].

There is an increased neural activation underlying the cognitive mechanisms associated with gratification upon talking about oneself. While people talked about themselves, brain activity was related to a pleasant experience compared to other natural rewards such as sex or food [8].

People’s “digital well-being” is the term used to refer to the impact of digital technologies on what it means to live a good life for a human being in various domains, in three broad themes such as positive computing, personalized human-computer interaction, and self-determination [9].

Collective environments exhibit the symptoms of such dependence, compromising people`s quality of life. The construction of a properly validated scale called the “Scale to assess leader`s perceptions about employees' digital addiction” (EPLDDE) contributes to studies on organizational functions, especially the quality of life [10].

In terms of commuting limitations, professional activity in the home office modality has become the “savior” of jobs, companies, and businesses, ensuring continuity of working remotely, even though not all people have ideal conditions to work at home. The lack of these conditions can, over time, have consequences on human behavior and cause changes in the general well-being of the subjects.

Pandemics and epidemics can affect people's physical and emotional health and disrupt society, usually resulting in high psychological distress and psychosocial maladjustment [11].

### 1.3 In the Social Sphere

A considerable current of researchers suggests that many intensive users of virtual devices develop dysfunctional symptoms that can severely affect functional and social areas of life [12].

Information technologies can change how we form relationships and socialize with those around us, with positive and negative effects depending on how we use or abuse them [13].

During the last decade, research on “addictive technological behaviors” showed a strong association between addictive use of technology and comorbid psychiatry disorders, confirming the relationship between symptoms of attention deficit (ADHD), anxiety, and depression could explain variance in addictive use of social networks and e videogames [12].

### 1.4 In the Professional Activities

There is a concern that digital dependency can reveal problems in organizations due to the demands of employee availability at all times, wherever they may be [12].

The world stage, with unlimited internet access by smartphone, has made social, cultural, and economic relations and has transformed the world to be faster and more efficient. Health professionals must be concerned about the majority of the patient`s care on the use of smartphones in the process. Not appropriating these devices in this job process may create severe impacts not only on the user`s life but also on community health care. It is necessary to watch how these professionals use smartphones during their work time [14].

Evidence suggests growth in preventing excessive use of smartphones and social media because their distraction affects academic and professional performance and productivity [15].

These habits are also in organizations and should be dealt with by Psychology and Psychiatry, given the requirements of companies that oblige the availability of employees at any time and place, so there is a need to take care of the health and well-being of their employees [16].

Many studies show high levels of dependence in health professionals who use their smartphones regularly during their clinical stage, which requires policies that restrict this use during work not to compromise the performance and the worker's well-being [17].

Changes in organizational relationships also influence relationships in these environments, transforming organizational culture.

## CONCLUSION

Impacts on professionals due to current organizational practices are facts demonstrated by research regarding physical and mental health. The perception of these facts is not always easy, but organizational leaders need to put this issue on their agendas.

## AUTHORS’ CONTRIBUTION

All authors participated in the conception of the article, in writing and reviewing

and gave final approval for the submitted manuscript.

## LIST OF ABBREVIATIONS

OCD: Obsessive-Compulsive Disorder
ADHD: Attention-Deficit

## References

[1] Bao Y, Sun Y, Meng S, Shi J (2020). 2019-nCOVID epidemic: address mental health care to empower society.. Lancet.

[2] Andreassen C.S., Billieux J., Griffiths M.D., Kuss D.J., Demetrovics Z., Mazzoni E., Pallesen S. (2016). The relationship between addictive use of social media and video games and symptoms of psychiatric disorders: A large-scale cross-sectional study.. Psychol. Addict. Behav..

[3] Gonçalves L.L., Nardi A.E., King A.L.S. (2020). Home Office in the COVID – 19 Pandemic: Impacts on Human Behavior..

[4] King A.L.S., Páua M.K., Guedes E., Nardi A.E. (2018). Digital Ergonomics..

[5] King A.L.S., Valença A.M., Silva A.C.O., Baczynski T., Carvalho M.R., Nardi A.E. (2013). Nomophobia: Dependency on virtual environments or social phobia?. Comput. Human Behav..

[6] Molina V.J.G. (2017). Luz azul: de las evidencias científicas a la atención al paciente..

[7] Brand M, Young KS, Laier C (2014). Prefrontal control and Internet addiction: a theoretical model and review of neuropsychological and neuro-imaging fundings.. Frontiers Hum. Neuroscienc.

[8] Guedes E., Sancassiani F. (2016). Internet Addiction and Excessive Social Network Use: What About Facebook?. Clin. Pract. Epidemiol. Ment. Health.

[9] Burr C, Taddeio M, Floridi L (2020). The etics of digital well-being: a thematic review.. Sci Eng. Ethics.

[10] Gonçalves L.L., Nardi A.E., Guedes E., Dos Santos H., Padua M.K., Guimaraes F.L., Rodrigues D., King A.L.S. (2018). Scale to assess Leadres‘perceptions about their workers’ digital addiction (EPLDDE).. Addict. Health.

[11] Zhang J., Wu W., Zhao X., Zhang W. (2020). Recommended psychological crisis intervention response to the 2019 novel coronavirus pneumonia outbreak in China: a model of West China Hospital.. Precis. Clin. Med..

[12] Gentile AD (2017). Internet gaming disorder in children and adolescents.. Pediatrics.

[13] Gokcearslam S., Mumai F.K., Haslaman T., Cevik Y.D. (2016). Modelagem do vício em smartphone: o papel do uso do smartphone, auto-regulação, auto-eficacia geral e cyberloafing on university students.. Computer Behav..

[14] King A.L.S., Pádua M.K., Gonçalves L.L., Santana de Souza Martins A., Nardi A.E. (2020). Smartphone use by health professionals: A review.. Digit. Health.

[15] Throuvala M.A., Griffiths M.D., Rennoldson M., Kuss D.J. (2020). Mind over Matter: Testing the Efficacy of an Online Randomized Controlled Trial to Reduce Distraction from Smartphone Use.. Int. J. Environ. Res. Public Health.

[16] Oliveira TS, Barreto LKS, El-Aonar WA, Souza LA, Pinheiro VLS (2017). Where`s my cell phone? Analysis of nomophobia in the organizational enviroment.. RAE – Revista de Administração de Empresas.

[17] Aguilera-Manrique G., Márquez-Hernandez V.V., Alcarez-Córdoba T., Granados-Gáriz G., Gutiérrez-Puertas V., Gutiérrez-Puertas L. (2018). The relation between Nomophobia and the distribution associated use of smartphone between nursing students in your clinical stage.. PLoS One.

